# Mitochondrial genomes of two eucotylids as the first representatives from the superfamily Microphalloidea (Trematoda) and phylogenetic implications

**DOI:** 10.1186/s13071-020-04547-8

**Published:** 2021-01-14

**Authors:** Suleman, Nehaz Muhammad, Mian Sayed Khan, Vasyl V. Tkach, Hanif Ullah, Muhammad Ehsan, Jun Ma, Xing-Quan Zhu

**Affiliations:** 1 State Key Laboratory of Veterinary Etiological Biology, Key Laboratory of Veterinary Parasitology of Gansu Province, Lanzhou Veterinary Research Institute, Chinese Academy of Agricultural Sciences, Lanzhou, Gansu 730046 People’s Republic of China; 2 Department of Zoology, University of Swabi, Swabi, Khyber Pakhtunkhwa Pakistan; 3 Department of Biology, University of North Dakota, Grand Forks, ND 58202-9019 USA; 4 Shanghai Veterinary Research Institute, Chinese Academy of Agricultural Sciences, Key Laboratory of Animal Parasitology, Shanghai, 20041 People’s Republic of China; 5 College of Veterinary Medicine, Shanxi Agricultural University, Taigu, 030801 Shanxi People’s Republic of China

**Keywords:** Microphalloidea, Eucotylidae, Mitochondrial genomes, Nucleotide diversity, Molecular phylogeny

## Abstract

**Background:**

The Eucotylidae Cohn, 1904 (Superfamily: Microphalloidea), is a family of digeneans parasitic in kidneys of birds as adults. The group is characterized by the high level of morphological similarities among genera and unclear systematic value of morphological characters traditionally used for their differentiation. In the present study, we sequenced the complete or nearly complete mitogenomes (mt genome) of two eucotylids representing the genera *Tamerlania* (*T. zarudnyi*) and *Tanaisia* (*Tanaisia* sp.). They represent the first sequenced mt genomes of any member of the superfamily Microphalloidea.

**Methods:**

A comparative mitogenomic analysis of the two newly sequenced eucotylids was conducted for the investigation of mitochondrial gene arrangement, contents and genetic distance. Phylogenetic position of the family Eucotylidae within the order Plagiorchiida was examined using nucleotide sequences of mitochondrial protein-coding genes (PCGs) plus RNAs using maximum likelihood (ML) and Bayesian inference (BI) methods. BI phylogeny based on concatenated amino acids sequences of PCGs was also conducted to determine possible effects of silent mutations.

**Results:**

The complete mt genome of *T. zarudnyi* was 16,188 bp and the nearly complete mt genome of *Tanaisia* sp. was 13,953 bp in length. A long string of additional amino acids (about 123 aa) at the 5′ end of the *cox*1 gene in both studied eucotylid mt genomes has resulted in the *cox*1 gene of eucotylids being longer than in all previously sequenced digeneans. The *rrnL* gene was also longer than previously reported in any digenean mitogenome sequenced so far. The TΨC and DHU loops of the tRNAs varied greatly between the two eucotylids while the anticodon loop was highly conserved. Phylogenetic analyses based on mtDNA nucleotide and amino acids sequences (as a separate set) positioned eucotylids as a sister group to all remaining members of the order Plagiorchiida. Both ML and BI phylogenies revealed the paraphyletic nature of the superfamily Gorgoderoidea and the suborder Xiphidiata.

**Conclusions:**

The average sequence identity, combined nucleotide diversity and Kimura-2 parameter distances between the two eucotylid mitogenomes demonstrated that *atp*6, *nad*5, *nad*4L and *nad*6 genes are better markers than the traditionally used *cox*1 or *nad*1 for the species differentiation and population-level studies of eucotylids because of their higher variability. The position of the Dicrocoeliidae and Eucotylidae outside the clade uniting other xiphidiatan trematodes strengthened the argument for the need for re-evaluation of the taxonomic content of the Xiphidiata.
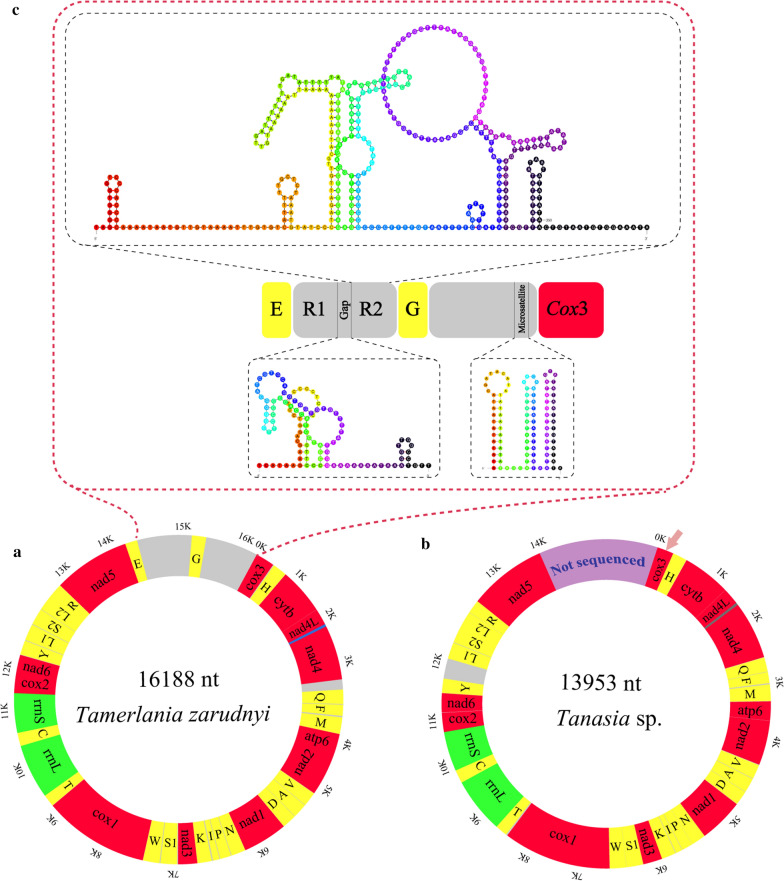

## Background

The Eucotylidae Cohn, 1904, is a group of digenetic renal flukes of birds reported from a diversity of birds worldwide [1, 2]. Eucotylids have a truncated heteroxenous life cycle with a single terrestrial pulmonate snail intermediate host; cercariae encyst within the sporocyst to form metacercariae [3, 4]. Birds are infected by ingesting molluscs containing metacercariae [3].

The system of the Eucotylidae has been unstable with several revisions utilizing somewhat different morphological characters as main diagnostic criteria [1, 5, 6]. Currently, the family contains two subfamilies, the Eucotylinae Skrjabin, 1924, and the Tanaisiinae Freitas, 1951 [1]. Species of the subfamily Tanaisiinae are characterized by the absence of the cervical thickening and cirrus sac and the presence of the seminal receptacle, intercaecal testes and caeca forming a cyclocoel [1, 6]. However, the key diagnostic characters of this subfamily were most recently amended by Lunaschi et al. [2] who described a well-developed cirrus sac with a small cirrus in a species of the Tanaisiinae. The three genera within the Tanaisiinae (*Tanaisia* Skrjabin 1924, *Tamerlania* Skrjabin 1924 and *Paratanaisia* Freitas, 1951) are differentiated by the extent of vitelline fields, the position of testes and the shape of tegumental scales or spines [1, 2, 5, 6]. However, the relative taxonomic value of these characters remains unclear and requires an independent set of characters. DNA sequences provide such an alternative source of data.

The taxonomic status of the genera *Tanaisia* and *Tamerlania* remained controversial for decades. Different authors either considered *Tamerlania* a separate genus or a synonym of *Tanaisia*, or its subgenus [5–7], mostly based on the similarities in the extent of the vitelline follicle fields. Although *Tamerlania* is currently recognized as a valid genus, some of its diagnostic characters are shared with both *Tanaisia* and *Paratanaisia* [1, 2]. Beside the problems at the genus level, the species boundaries within this group of renal flukes also remain uncertain. Therefore, DNA sequences, especially from fast-mutating genes, are critically important for addressing existing problems of eucotylid evolutionary interrelationships and taxonomy.

Previous molecular phylogenetic studies based on nuclear ribosomal DNA (rDNA) included very few representatives of the Eucotylidae in order to position the family within higher taxa [8–12]. They invariably placed the Eucotylidae within the suborder Xiphidiata as a long-branched clade. However, as mentioned above, more variable molecular markers are needed to re-evaluate the relative weight of morphological criteria traditionally used in eucotylid systematics to solve questions of differentiation among genera and species within this group, while more genes in general need to be used to examine its relationships with other digeneans.

Mitochondrial (mt) genomes represent a rich source of genetic markers with greater variability and have been widely used in parasitic flatworm population genetics and systematics [13–18]. The growing amount of mitogenome data in GenBank improves its utility in molecular phylogenetics of trematodes. However, mitogenome data remain very scarce or completely lacking for many groups of trematodes including the Eucotylidae and the whole superfamily Microphalloidea. To at least partially fill this gap, we have sequenced and annotated complete mitogenome of *Tamerlania zarudnyi* Skrjabin, 1924 (type species of the genus *Tamerlania*), and nearly complete mitogenome of *Tanaisia* sp. collected from birds in Pakistan. The aims of this study were to: (i) characterize and compare the previously unstudied mitogenomes of eucotylids; (ii) explore the phylogenetic relationships among currently sequenced eucotylid genera; (iii) test the monophyly of the suborder Xiphidiata Olson, Cribb, Tkach, Bray & Littlewood, 2003, using available mitogenome data.

## Methods

### Specimen collection, morphological examination and genomic DNA isolation

Twenty adult specimens of *T. zarudnyi* Skrjabin, 1924, were collected from kidneys of the house crow (*Corvus splendens*) and 15 specimens of *Tanaisia* sp. were collected from the kidney of the little ringed plover (*Charadrius dubius*) (Charadriiformes: Charadriidae) in the Swabi district, Khyber Pakhtunkhwa province, Pakistan. Trematode specimens were preserved in 80% ethanol and stored in a freezer [19]. Two digenean specimens from each host were processed for morphological studies according to the protocol recommended by Lutz et al. [19] and identified to species or merely to genus level using published descriptions and keys [1, 2, 5, 6, 20, 21]. For molecular studies, total genomic DNA (gDNA) of three specimens from each species was extracted from ethanol-preserved specimens using Wizard^®^ SV Genomic DNA Purification System (Promega, Madison, WI, USA) according to the protocol described by Gasser et al. [22] following the manufacturer’s instructions.

### Amplification and analysis of nuclear ribosomal DNA

For the taxonomic identification, the nuclear rDNA ITS region was amplified with the primers BD1and BD2 [23], and to examine the phylogenetic interrelationships among eucotylid genera, D1–D3 region of the large subunit (LSU) of rDNA (28S rDNA) was amplified utilizing the primers LSU5 (forward) and 1500R (reverse) [9, 24] as described in our previous studies [25, 26]. To determine intraspecific differences, DNA of three specimens from each species was used separately for the amplification of each marker. All resultant positive PCR amplicons were purified using EZNA Gel Extraction Kit (OMEGA Bio-tek Inc., Doraville, GA, USA) and sent to Genewiz Company (Beijing, China) for sequencing. The obtained nucleotide sequences for each marker were assembled using DNAstar v7.1 [27] and Clustal X 1.83 [28] software. Sequence identity (%) across the ITS rDNA region among newly obtained sequences and another eucotylid, *T*. *valida*, the only presently available ITS rDNA of eucotylid in NCBI GenBank, was calculated using BioEdit 7.0.9.0 [29]. Similarly, prior to phylogenetic analysis, sequence identity across the D1–D3 region of LSU among newly sequenced and four other eucotylids, presently available in GenBank, was also determined.

To assess the phylogenetic interrelationships of our specimens within the family Eucotylidae, the newly obtained 28S rDNA sequences were aligned with available sequences of other eucotylid species, using MEGA X [30]. *Renicola* sp. was used as the outgroup based on the results of previous studies suggesting the close relationships of Eucotylidae and Renicolidae [8, 9]. The resulting alignment, trimmed to the length of the shortest sequence, was 910 bp long, including a few small gaps due to indels.

Phylogenetic analyses were conducted using Bayesian inference (BI) as implemented in MrBayes version 3.2.6 software [31, 32]. The GTR+G+F model was identified as the best fitting nucleotide substitution model using jmodeltest 2 software [33]. BI analysis was performed using MrBayes software as follows: two parallel Markov chain Monte Carlo (MCMC) chains were run for 10,000,000 generations. The initial 25% of sampled data generated was treated as “burn-in”, and the final 75% of trees was used for calculating Bayesian posterior probabilities (BPP). The phylograms were visualized in FigTree version 1.4 software [34] and annotated in Adobe Illustrator^®^.

### Long PCR-based sequencing of eucotylid mt genomes

Sequences of short mitochondrial genome fragments (*cox*3-*cytb*, *rrnL*-*rrnS*) and partial genes (*nad*4, *nad*1, *cox*1, *nad*5) were obtained using platyhelminth universal primers [35]. The obtained sequences were further used to design six or five pairs of species specific primers (Additional file 1: Table S1) for the amplification of complete or nearly complete mt genomes of our eucotylids in medium to long overlapping fragments. Long mt genome fragments (2.5–3.5 kb) were amplified by long-PCR reactions using PrimeStar Max DNA polymerase premix (Takara, Dalian, China) following the procedure described in our previous studies [25, 26] and sequenced directly by Genewiz sequencing company (Beijing, China) using the primer-walking strategy.

### mtDNA genomes assembly, annotation and analyses

The obtained sequences were carefully examined by checking chromatograms for quality and double peaks following BLASTn analysis to make sure that all amplicons are the desired target sequences. DNA extracted from a single individual of each species was used to infer and annotate its mitogenome, thereby avoiding any intraspecific variation. Eucotylid mtDNA sequences were assembled using DNAstar v.7.1 software [27] and aligned against selected digenean mitogenomes and then against each other using MAFFT 7.149 [36] to determine the relative positions of genes. Boundaries of protein-coding genes (PCGs) were found by searching for open reading frame (ORF; NCBI) and checking alignment against the selected digenean mitogenomes. tRNA sequences and their secondary structure were identified using the MITOS [37] and ARWEN [38] web servers. The two ribosomal RNAs, *rrnL* and *rrnS*, were determined via a comparison with the mt genome sequences of selected trematode species and their boundaries were assumed to extend to their adjacent genes. The nucleotide identity (%), nucleotide and amino acid composition, A+T/G+C skewness, codon usage and relative synonymous codon usage (RSCU) for PCGs were determined in PhyloSuite v1.2.1 [39]. The stacked bar chart of amino acids used for the construction of mt PCGs was drawn using ggplot2 [40]. DnaSP v.6 [41] was used to conduct the sliding window analysis: window size of 200 bp and a step size of 20 bp were implemented to estimate the nucleotide divergence (Pi) among PCGs, rRNAs and tRNAs of the three eucotylid mitogenomes. Kimura-2-parameter (K2P) genetic distances of the mt PCGs (substitution included = transitions + transversions) were also calculated using MEGA X [30]. To detect tandem repeats of nucleotides in the two non-coding regions of the complete mt genome of *T. zarudnyi*, we used Tandem Repeat Finder [42] and mreps [43] software. The circular diagram of the mt genomes was drawn with MTVIZ, an online tool of mitochondrial visualization (available at: (http://pacosy.informatik.uni-leipzig.de/mtviz/).

### Phylogenetic analyses based on mt genomes

Phylogenetic analyses were conducted using the two newly sequenced eucotylid mitogenomes and 28 other selected trematodes mitogenomes of the order Plagiorchiida La Rue, 1957. *Schistosoma japonicum* belonging to the order Diplostomida Olson, Cribb, Tkach, Bray, and Littlewood, 2003, was used as the outgroup. Two datasets were processed for phylogenetic analyses: dataset 1 containing nucleotide alignment of 11 PCGs, 2 rRNAs and 20 tRNAs and dataset 2 containing amino acid alignment of 11 PCGs. The *trnG*, *trnE* and *cox*3 were excluded from analyses because we were unable to obtain complete sequences of these genes for *Tanaisia* sp. The PhyloSuite program [39] was used to generate GenBank files of the studied eucotylids. Fasta files with nucleotide sequences of PCGs, rRNAs and tRNAs were extracted from the GenBank files. The nucleotide sequences of PCGs were translated to their corresponding amino acids using PhyloSuite. Alignments of both datasets were performed separately using the MAFFT program [36] integrated in PhyloSuite, wherein for dataset 1, codon-alignment mode was used for PCGs, and normal alignment mode was used for the RNA nucleotide sequences. The alignments were then concatenated (each as a separate set) in PhyloSuite after the removal of ambiguously aligned regions using Gblocks 0.91b [44]. The resulting files were then subjected to ModelFinder [45] to find the most appropriate evolutionary models for both datasets to conduct maximum likelihood (ML) and Bayesian inference (BI) methods of phylogenetic analyses.

For dataset 1, maximum likelihood phylogeny was inferred using IQ-TREE [46] with the GTR+R5+F model as the best fit model of nucleotide substitution by performing ultrafast bootstraps [47] with 100,000 replicates. BI phylogeny was inferred using MrBayes 3.2.6 [31, 32] (with default settings) under the GTR+I+G+F model using two MCMC chains for 10,000,000 generations and 1000 sample frequency, in which the initial 25% of trees was discarded as ‘burn-in’ and the rest was used to calculate the BPP.

To remove effects of possible mutation saturation due to silent mutations, dataset 2 (alignment of translated sequences of 11 PCGs) was analyzed in MrBayes with the Jones+I+G+F model as the best fitting model of amino acid evolution. The analysis was conducted using the same parameters as described above for the nucleotide-based BI phylogeny. The phylograms were visualized and annotated in iTOL [48] and Adobe Illustrator^®^.

## Results and discussion

### Comparison of nuclear rDNA markers and phylogenetic relationships within Eucotylidae

**Fig. 1:**
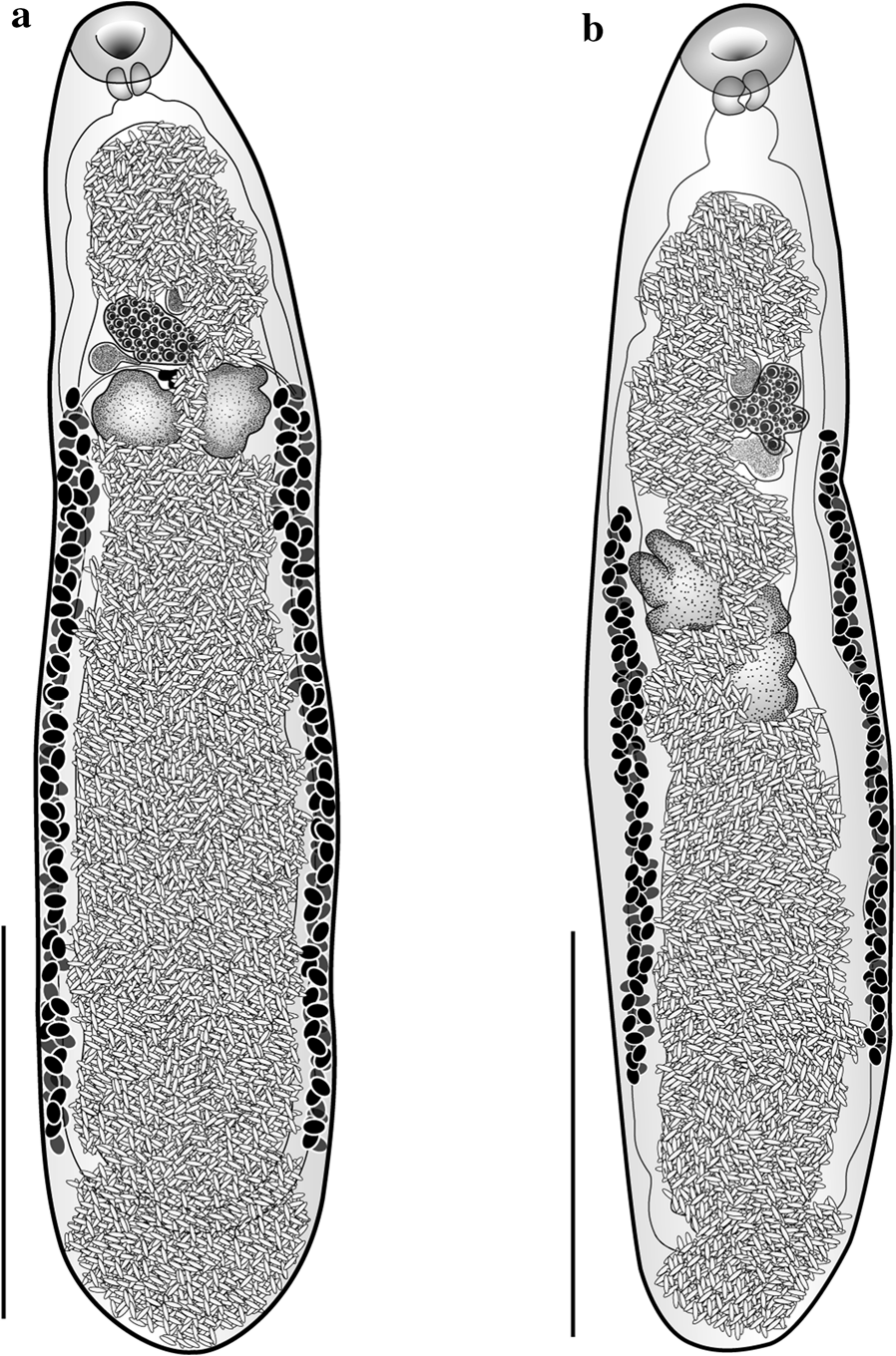
General view of the studied eucotylids. **a** *Tamerlania zarudnyi* **b** *Tanaisia* sp. Scale bars: 1 mm

No intraspecific variation was observed in the nucleotide sequences of both markers sequenced from three specimens of each species in this study. The examined eucotylid collected from *C. splendens* agreed with the key features of the genus *Tamerlania* and all qualitative and morphometric characteristics of *T. zarudnyi* [20] (Table 1: Fig. 1a). Moreover, the 28S rDNA sequences (1255 bp; GenBank accession no. MW131090) showed 99.52% identity with corresponding sequences presently available in GenBank for this species. The minor 28S sequence variability can be explained by the substantial geographic distance between the collection localities of the two sequenced samples. It is worth noting that we collected our material in the region that is relatively close to the area where the species was originally described [49]. The ITS rDNA region of *T. zarudnyi* was 946 bp in length (GenBank accession no. MW159308); to date, no other *Tamerlania* sequences are available in GenBank.

**Table 1:** Comparative measurements of *Tamerlania zarudnyi* (present study) and *Tanaisia* sp. with *Tamerlania zarudnyi* (previously published) and *Tanaisia fedtschenkoi*, respectively

Characters	*Tamerlania zarudnyi* (present study), Pakistan	*Tamerlania zarudnyi* (Byrd and Denton, 1950), USA	*Tanaisia* sp. (Present study), Pakistan	*Tanaisia fedtschenkoi* (Byrd and Denton, 1950), USA
Total body length	3.0–3.7	1.94–3.23	2.41-–3.35	1.62–3.46
Body maximum width	0.77–0.86	0.40–0.66	0.49–0.74	0.41–0.71
Oral sucker length	0.19–0.21	0.13–0.29	0.14–0.21	0.13–0.22
Oral sucker width	0.20–0.27	0.16–0.32	0.2–0.29	0.17–0.29
Pharynx length	0.09–0.10	0.06–0.12	0.08–0.09	0.05–0.09
Pharynx width	0.11–0.13	0.07–014	0.10–0.12	0.08–0.14
Oesophagus length (μm)	24–40	42	90-109	34–93
Cirrus sac length (μm)	80–90	60–85	82-110	? –85
Ovary length	0.17–0.18	0.08–0.21	0.17–0.21	0.15–0.2
Ovary width	0.21–0.26	0.13–0.23	0.19–0.21	0.14–0.2
Anterior (right) testis length*	0.19–0.25	0.07–0.20	0.21–0.22	0.15–0.2
Anterior (right) testis width*	0.21–0.23	0.07–0.22	0.20–0.22	0.10–0.2
Posterior (left) testis length*	0.20–0.24	0.08–0.20	0.23–0.35	0.13–0.3
Posterior (left) testis width*	0.18–0.23	0.08–0.20	0.18–0.21	0.10–0.2
Unity of caeca from posterior end	0.25–0.47	–	0.29–0.33	–
Ending of vitellria from posterior end	0.42–0.98	0.43–1.06	0.90–0.97	–
Eggs length (μm)	32-35	32–50	32–34	33–38
Eggs width (μm)	16-21	25–32	11–17	10–19

*Anterior/posterior in case of *Tanaisia* sp. and right/left in case of *Tamerlania zarudnyi*

The ITS rDNA region of our *Tanaisia* sp. (935 bp; GenBank accession no. MW159299) showed 96.36–97.64% identity with sequences of *T*. *valida* (KX913703–X913711) deposited in the GenBank by authors from Brazil (unpublished). The same authors also deposited 28S rDNA sequences of their two samples of *T. valida*, Tv6 (1352 bp; KX913713) and Tv12 (1245 bp; KX913714). These samples demonstrated only 95.02% identity with each other, which indicates that the sequences submitted to GenBank as two isolates of *T. valida* represent two different species. At the same time, the differences between the two Brazilian samples were observed only at the 5′ and 3′ ends of their partial 28S sequences which suggests a problematic quality of sequences at both ends of either both samples or one of them. Upon removal of the potentially problematic 280 bp from the 5′ end and 63 bp from the 3′ end, the remaining 902 bp was 100% identical. Therefore, we used only this 902 bp of 28S rDNA of *T*. *valida* in our phylogenetic analysis of the Eucotylidae and trimmed the remaining sequences to the same length.

**Fig. 2:**
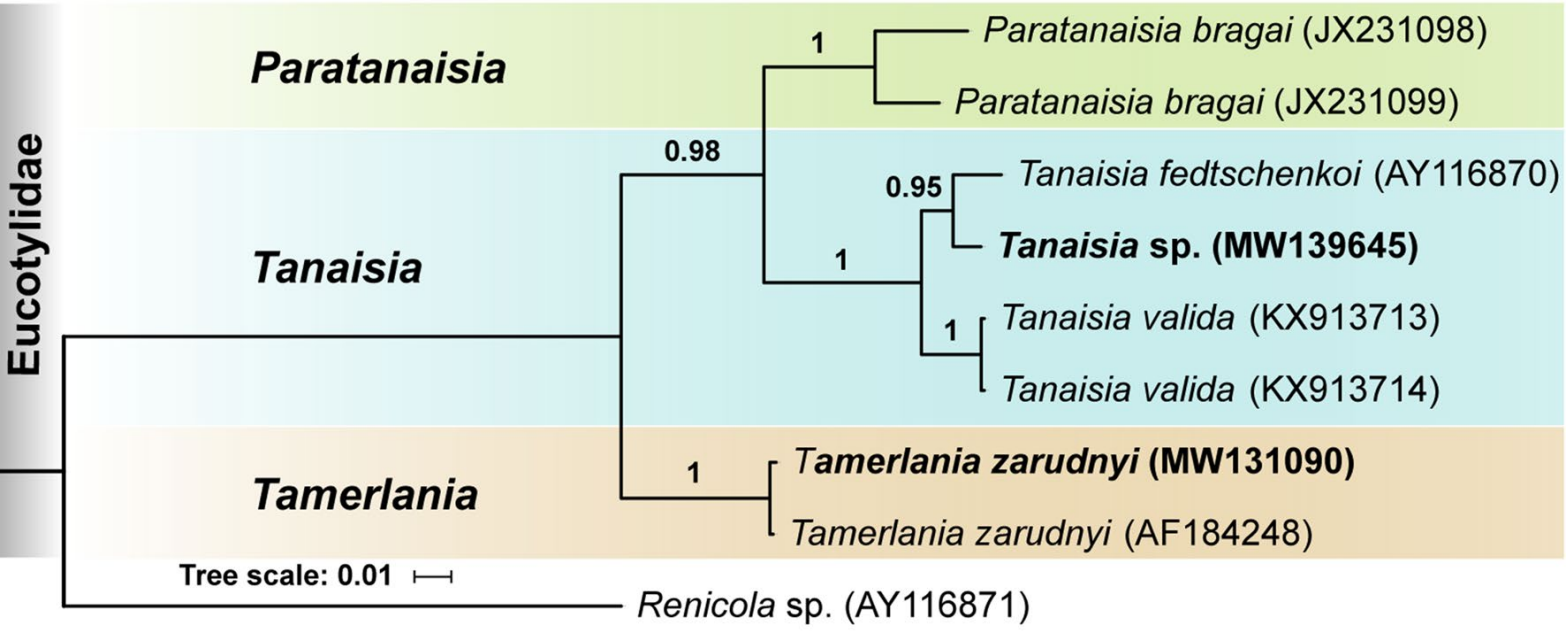
Phylogenetic interrelationships among eucotylids resulting from Bayesian inference (BI) analysis using partial 28S rDNA sequences. Node labels indicate Bayesian posterior probabilities (> 90%). New sequences generated in this study are in bold. Sequence of *Renicola* sp. is used as outgroup

A preliminary analysis of the position of the Eucotylidae (including our new sequences) among plagiorchiatan digeneans (not shown) did not show any differences compared to previously published phylogenies [8, 9, 12]. Our BI analysis of the interrelationships within the Eucotylidae based on partial 28S rDNA sequences placed the two sequences (one previously available and our new sequence) of *T. zarudnyi* together in a clade with 100% nodal support (Fig. 2). Our specimens identified as *Tanaisia* sp. clustered together with *T. fedtschenkoi* with high nodal support (BPP = 0.95), which was expected because the sequenced sample of *T. fedtschenkoi* originated from Europe and *T. valida* was collected in South America. 28S rDNA sequences of our *Tanaisia* sp. (GenBank accession no. MW139645) showed 98.49% identity with corresponding sequences of *T. fedtschenkoi* (1255 bp; AY116870). Although the key morphological features and dimensions of *Tanaisia* sp. corresponded to those of *T. fedtschenkoi* [20] (Table 1; Fig. 1b), considering the level of divergence in 28S sequences and the lack of ITS rDNA or mitochondrial sequences of *T. fedtschenkoi* in the GenBank, we opted to identify our *Tanaisia* sp. to the genus level only.

Despite the limited amount of currently available sequence data, all three recognized genera of the Tanaisiinae are represented in the phylogeny. Importantly, the type species of all three genera have been sequenced. This provides an opportunity to make some preliminary conclusions regarding the relative value of morphological criteria used in the systematics of the Tanaisiinae. The phylogenetic analysis clearly indicates that *Tanaisia* is phylogenetically closer to *Paratanaisia* than to *Tamerlania* (Fig. 2). The most obvious morphological character shared between the two genera is the relative position of the testes, which are tandem or nearly tandem in *Tanaisia* and *Paratanaisia*, but symmetrical in *Tamerlania*. At the same time, several previous authors considered *Tamerlania* a synonym or a sub-genus of *Tanaisia* [5–7], mostly based on the similarity in the extent of the fields of vitelline follicles, which do not reach posterior margin of the ovary on both genera. In *Paratanaisia* the vitelline fields extend much further anteriorly and reach the level of intestinal bifurcation [1]. Thus, molecular phylogeny provides evidence that the relative position of testes is more indicative of close evolutionary relationships among taxa of Tanaisiinae than the position of vitelline fields. Although both characters seem to be useful for the practical purpose of differentiation among genera, it is clear that more sequence data with denser taxonomic and geographic sampling are necessary for more definitive conclusions.

### Eucotylids mitochondrial genome and base composition

The complete mitogenome of *T. zarudnyi* (GenBank accession no. MW334947) was 16,188 bp long and the partial mitogenome of *Tanaisia* sp. (GenBank accession no. MW334948) was 13953 bp in length. The mitogenome of *T. zarudnyi* was one of the largest mt genomes among trematodes sequenced so far, next to *Echinostoma paraensei* (20,298 bp; KT008005; direct GenBank submission), *E. revolutum* (17,030 bp; MN496162; [50]) and *Schistosoma spindale* (16,901 bp; DQ157223; [51]).

We were unable to amplify the fragment containing non-coding regions (partial *trn*E-LNCR-*trn*G-SNCR-partial *cox*3) of the mt genomes of *Tanaisia* sp. This was likely due to the presence of repetitive AT-rich sequences resulting in difficulties in PCR amplification. Similar region(s) in other studies of mitochondrial genomes of flatworms have also been shown as problematic [51–54]. This is a more general issue with the Sanger sequencing, which is often unable to sequence complete non-coding region(s) of mt genomes having several tandem repeats [54]. Recently, the PacBio single-molecule real-time sequencing method was used to characterize long and complicated repetitive regions of flatworm mitogenomes [54, 55]. Nonetheless, the nearly complete mt genome of *Tanaisia* sp. sequenced in the present study proved to be sufficient for phylogenetic reconstructions and a variety of comparative analyses.

**Fig. 3:**
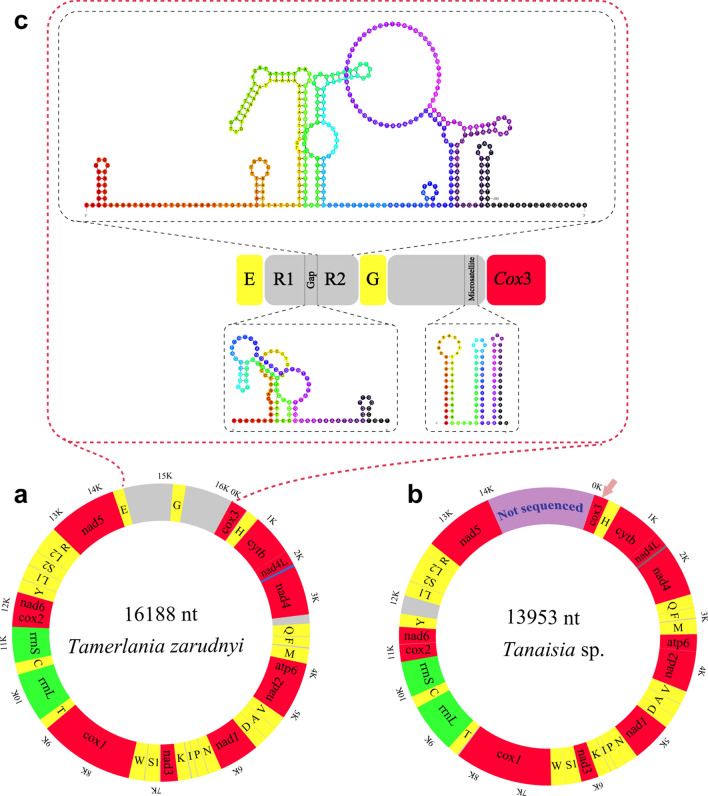
Organization of the mitochondrial genome of the two eucotylids (**a**, **b**) with secondary structures of one set of repeated sequences and gap between them present in the large non-coding regions (LNCR) and short microsatellite in the short non-coding region (SNCR) of *Tamerlania zarudnyi* (**c**). Grey colour indicates the intergenic sequences and NCRs. Arrow in (**b**) denotes the partial sequences of *cox*3 gene of the mitogenome of *Tanaisia* sp.

The mt genomes of both sequenced eucotylids lacked the *atp*8 gene and all the genes were encoded on the same strand (H strand). Genes were either separated by non-coding intergenic sequences (1–320 bp), located immediately one after another or even overlapped by 1–40 bp. The general architecture and comparison of orthologous sequences for the two studied eucotylid mitogenomes are summarized in Table 2. The complete circular mt genome of *T. zarudnyi* (Fig. 3a) contained the typical flatworm 36 mitochondrial genes with overall A–T content of 60.5%. The A–T content of the partial mt genome of *Tanaisia* sp. was 56.7%. The A–T content in both eucotylid mitogenomes was within the range observed in other trematode mitogenomes including xiphidiates, e.g. 51.7% in *P. westermani* (AF219379; direct GenBank submission) and 65.24% in *Plagiorchis maculosus* (MK641809; [25]). The nucleotide composition and skewness in overall mitogenomes, individual genes and each codon position (1st, 2nd, 3rd) of the studied eucotylids are listed in the table (Additional file 2: Table S2).

**Table 2:** Comparison of the annotated mitochondrial genomes of *Tamerlania zarudnyi* and *Tanaisia* sp.

Gene	Position		Size (bp)	IGN	Codon		Identity (%)
	From	To			Start	Stop	
*Tamerlania zarudnyi*/*Tanaisia* sp.							
*cox*3	1/1	651/120	651/120		ATG/CGG	TAG/TAG	13.12
*trnH*	667/123	734/192	68/70	15/2			66.2
*cytb*	738/198	1850/1310	1113/1113	3/5	GTG/GTG	TAG/TAG	71.61
*nad*4L	1853/1313	2122/1585	270/273	2/2	GTG/ATG	TAA/TAG	63
*nad*4	2083/1546	3366/2850	1284/1305	−40/−40	ATG/ATG	TAG/TAG	65.44
*trnQ*	3529/2857	3593/2922	65/66	162/6			66.67
*trnF*	3604/2930	3669/2994	66/65	10/7			78.79
*trnM*	3692/3014	3762/3083	71/70	22/19			73.61
*atp*6	3765/3089	4301/3622	537/534	2/5	ATG/ATG	TAG/TAG	46.95
*nad*2	4301/3626	5170/4492	870/867	–1/3	GTG/ATG	TAG/TAA	69.31
*trnV*	5176/4502	5241/4567	66/66	5/9			68.18
*trnA*	5249/4577	5318/4646	70/70	7/9			84.29
*trnD*	5325/4653	5392/4716	68/64	6/6			61.76
*nad*1	5396/4722	6292/5639	897/918	3/5	ATG/GTG	TAG/TAG	75.05
*trnN*	6295/5642	6364/5707	70/66	2/2			64.29
*trnP*	6375/5711	6440/5778	66/68	10/3			75
*trnI*	6452/5786	6521/5852	70/67	11/7			78.57
*trnK*	6541/5863	6613/5929	73/67	19/10			63.01
*nad*3	6616/5930	6972/6280	357/351	2/–	ATG/GTG	TAG/TAG	68.91
*trnS*1	6987/6283	7047/6344	61/62	14/2			54.84
*trnW*	7062/6348	7130/6414	69/67	14/3			69.57
*cox*1	7134/6418	9188/8478	2055/2061	3/3	ATG/GTG	TAG/TAG	69
*trnT*	9193/8502	9257/8571	65/70	4/23			74.29
*rrnL*	9258/8572	10501/9802	1244/1231				73.3
*trnC*	10502/9803	10570/9868	69/66				74.29
*rrnS*	10571/9869	11328/10645	758/777				80.76
*cox*2	11329/10646	11925/11245	597/600		GTG/GTG	TAG/TAG	74.5
*nad*6	11926/11251	12375/11697	450/447	–/5	GTG/GTG	TAG/TAG	59.56
*trnY*	12382/11701	12446/11764	65/64	6/3			73.13
*trnL*1	12457/12085	12521/12150	65/66	10/320			79.1
*trnS*2	12532/12160	12604/12228	73/69	10/9			61.64
*trnL*2	12611/12229	12679/12297	69/69	6/–			78.57
*trnR*	12679/12302	12746/12364	68/63	–1/4			64.71
*nad*5	12749/12367	14344/13953	1596/1587	2/2	ATG/ATG	TAG/TAG	60.53
*trnE*	14344/–	14411/–	68/–	–1/–			
LNCR	14412/–	15286/–	875/–				
*trnG*	15287/–	15353/–	67/–				
SNCR	15354/–	16188/–	835/–				

bp: base pairs; IGN: intergenic nucleotide; LNCR: large non-coding region; SNCR: short non-coding region

### Protein-coding genes and codon usage

The shortest protein-coding gene in the mt genomes of the studied eucotylids was *nad*4L (270–273 bp), whereas the longest was *cox*1 (2055–2061 bp). Notably, the size of the *cox*1 gene in eucotylid mt genomes was the largest among all currently sequenced digeneans. This is due to the presence of a long additional amino acid tract (about 123 aa) at the 5′ end of the *cox*1 gene in both studied eucotylids. The size of the *cox*1 gene reported from the majority of plagiorchiidan trematodes varies from 1533 bp in *Fasciola hepatica* [56, 57] to 1567 bp in *Plagiorchis maculosus* [25]. However, in the mt genomes of diplostomidan species, the size of the *cox*1 gene is significantly longer and ranges from 1611 bp in *Cotylurus marcogliesei* [18] to 1830 bp in *Schistosoma mansoni* [58], which has 60 additional amino acids at the start of the *cox*1 gene.

All PCGs of the studied eucotylid mt genomes used ATG or GTG as start codons, whereas the most frequent stop codon was TAG (for 11/12 PCGs in each species) followed by TAA used for *nad*4L in *T. zarudnyi* and for *nad*2 in *Tanaisia* sp. (Table 2). The abbreviated or incomplete stop codons T or TA were not used in these mitogenomes. The most frequently used codons in mitochondrial PCGs of both eucotylids were UUU, UUG and GUU, while the least used codons were ACA, CAA and AAC. Codon CAA was entirely missing in PCGs of *Tanaisia* sp. Thus, codons ending in U or G were predominant (≥ 83%), resulting in high AT skewness in the third codon position (− 0.682 in *T. zarudnyi* and − 0.768 in *Tanaisia* sp.) (Additional file 2: Table S2). Similarly, leucine (L1 + L2), valine, phenylalanine and glycine were the most frequent amino acids in the PCGs of the eucotylid mitogenomes, which were also observed in other xiphidiates [25, 26, 59–61]. Codon usage and relative synonymous codon usage (RSCU) of both eucotylids mitogenomes are presented here (Additional file 3: Figure S1).

### Transfer and ribosomal RNA genes

All 22 tRNA genes found in the complete mtDNA of *T. zarudnyi* ranged in length from 61 bp (*trnS*1) to 73 bp (*trnK*) (Table 2). Sequences of two tRNA genes, *trn*E and *trn*G, were not obtained for the mt genomes of *Tanaisia* sp. whereas the remaining 20 tRNAs ranged from 62 bp (*trnS*1) to 70 bp (*trnH*, *trnM*, *trnA* and *trnT*) in *Tanaisia* sp. All tRNA sequences could be folded into the typical cloverleaf structure, except *trnS*1, which lacked the dihydrouridine (DHU) arm in both eucotylid mitogenomes (Additional files 4 and 5: Figure S2 and S3). Similarly, standard anticodons were found in all tRNAs of these eucotylid mitogenomes. The nucleotide identity and substitution model among the tRNAs of the two eucotylid mitogenomes are also presented (Additional file 4 and 5: Figure S2 and S3). The TΨC and DHU loops were highly variable while the anticodon loop was highly conserved with only one (*trnH*, *trnV*, *trnI*) or two (*trnK*) nucleotide substitutions. We also found the non-canonical or non-Watson-Crick pairs in different stems (acceptor, anticodon, TΨC and DHU), as commonly found in other trematode mitogenomes [16, 55, 62]. G–T was the most common non-canonical base pair in the DHU stem of tRNAs in both eucotylids. The nucleotide substitutions among different stems were mostly compensatory or hemi-compensatory base changes (G–T ↔ A–T and C–A to T–A) where there was a single nucleotide mutation in a base pair maintaining their bond in the mitochondrial tRNA of other species.

The large subunit of mitochondrial rRNA (*rrnL*) was 1244 bp long in *T. zarudnyi* and 1231 bp long in *Tanaisia* sp., which is longer than previously reported in any other trematode mitogenomes. The size of the small rRNA subunit (*rrnS*) was within the range reported for other trematode mitogenomes (Table 2). *rrnL* and *rrnS* were separated by *trnC*. Both eucotylid mitogenomes had shared gene boundaries in *trnT*-*rrnL*-*trn*C-rrnS, which has been observed in nearly all flatworms characterized so far.

### Non-coding regions and intergenic sequences

Apart from short intergenic sequences (1–23 nt), one long stretch of intergenic sequences of 162 nt between *nad*4 and *trnQ* in the mt genomes of *T. zarudnyi* and 320 nt between *trnY* and *trnL*1 in the mt genome of *Tanaisia* sp. were also found. The complete mitogenome of *T. zarudnyi* contains two non-coding regions (NCRs): a large non-coding region (LNCR; 875 bp) and a short non-coding region (SNCR; 835 bp). Both NCRs are located at the usual positions reported in other digeneans (between *trnG* and *cox*3) and separated by *trnE*. The LNCR contains two sets of identical sequences (367 bp each), separated from each other by a stretch of 111 nucleotides. Each set of sequences as well as the gap between them is capable of forming putative secondary structures containing several stem loops (Fig. 3c). Microsatellite-like simple sequence repeats (SSRs) of TA_52_ were also found in the SNCR of *T. zarudnyi.* These microsatellite-like sequences can be folded in three stem-loop structures where the A–T bond is replaced by A–A or G–A at three positions (a single instance in each stem).

### Sliding window analysis and nucleotide diversity

**Fig. 4:**
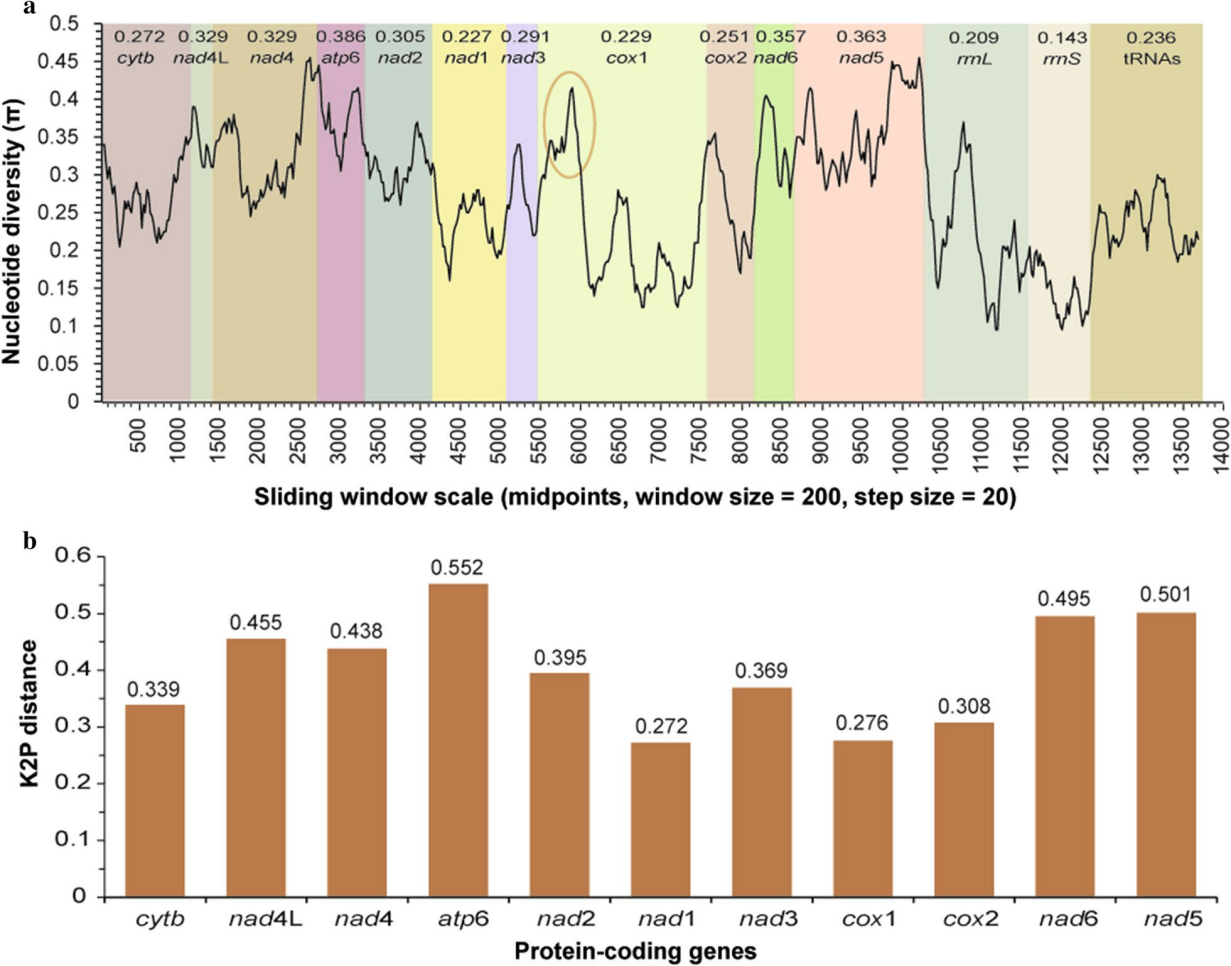
Comparative analyses of two eucotylid mitogenomes. **a** Sliding window analysis of the alignment of 11 protein-coding genes (PCGs), 2 rRNAs and 18 coalescent tRNAs (*trn*E and *trn*G are omitted as we were unable to obtain their sequences for one of the species). The black line represents nucleotide variation in a window of 200 bp (step size = 20 bp, with the values inserted at its mid-point). Gene boundaries are indicated by colour with mean variation ratio per gene shown above each gene. The region indicated by the circle in *cox*1 gene shows nucleotide variation in the additional sequences at the 5′ end of this gene in both studied eucotylid mt genomes **b** The Kimura-2-parameter distance (K2P) among 11 PCGs of eucotylid mitogenomes

The sliding window analysis (window size = 200, step size = 20) of the aligned mitogenomic sequences of the two studied eucotylids showed relatively low nucleotide diversity (Pi values) of *rrnS* (0.143), *rrnL* (0.209), *nad*1 (0.227), *cox*1 (0.229), *cox*2 (0.251) and *cytb* (0.272) while genes with relatively high nucleotide diversity included *atp*6 (0.386), *nad*5 (0.363), *nad*6 (0.357) and *nad*4L and *nad*4 (0.329 each) (Fig. 4a). A similar result was obtained from the Kimura-2-parameter (K2P) genetic distance analysis of 11 PCGs (substitutions included = transitions + transversions) where *nad*1, *cox*1, *cox*2 and *cyt*b had the lowest K2P genetic distances, while *atp*6, *nad*5, *nad4*L and *nad*6 had comparatively high K2P genetic distances (Fig. 4b). These analyses and average sequence identity (Table 2) consistently indicated that *atp*6, *nad*5, *nad*4L and *nad*6 are fast-evolving genes in the two eucotylid mitogenomes.

Similarly high levels of nucleotide variation in *atp*6, *nad*5 and *nad*6 genes have been reported in mt PCGs of a variety of flatworms [63–65]. Since hypervariable genes are more suitable to resolve recently diverged lineages [66], we consider the fast-evolving *atp*6, *nad*5 and *nad*6 to be suitable molecular markers for identification, systematics and population level studies in the family Eucotylidae.

### Phylogeny of xiphidiatan trematodes and other selected trematodes based on mt genomes

**Fig. 5:**
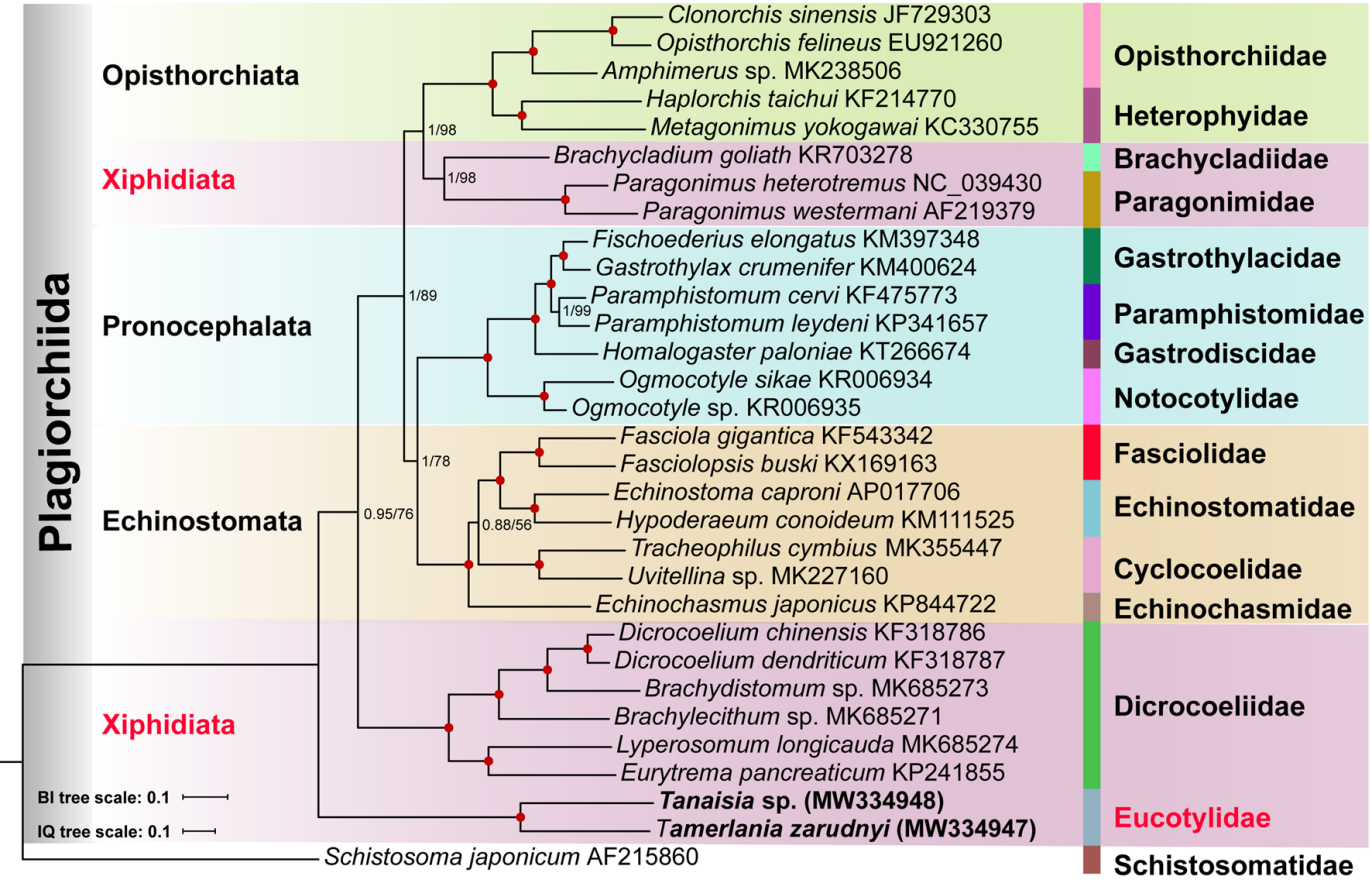
Phylogeny of the order Plagiorchiida based on Bayesian inference (BI) and maximum likelihood (ML) analyses using concatenated nucleotide sequences of protein-coding genes, rRNAs and tRNAs of 28 xiphidiatan mitogenomes. Statistical support values (bootstrap/Bayesian posterior probability) of ML/BI analysis are shown at the nodes. Circles indicate ML/BPP = 100/1.0; other values are given at the nodes. Suborders and families are highlighted by individual colours. *Schistosoma japonicum* is the outgroup

**Fig. 6:**
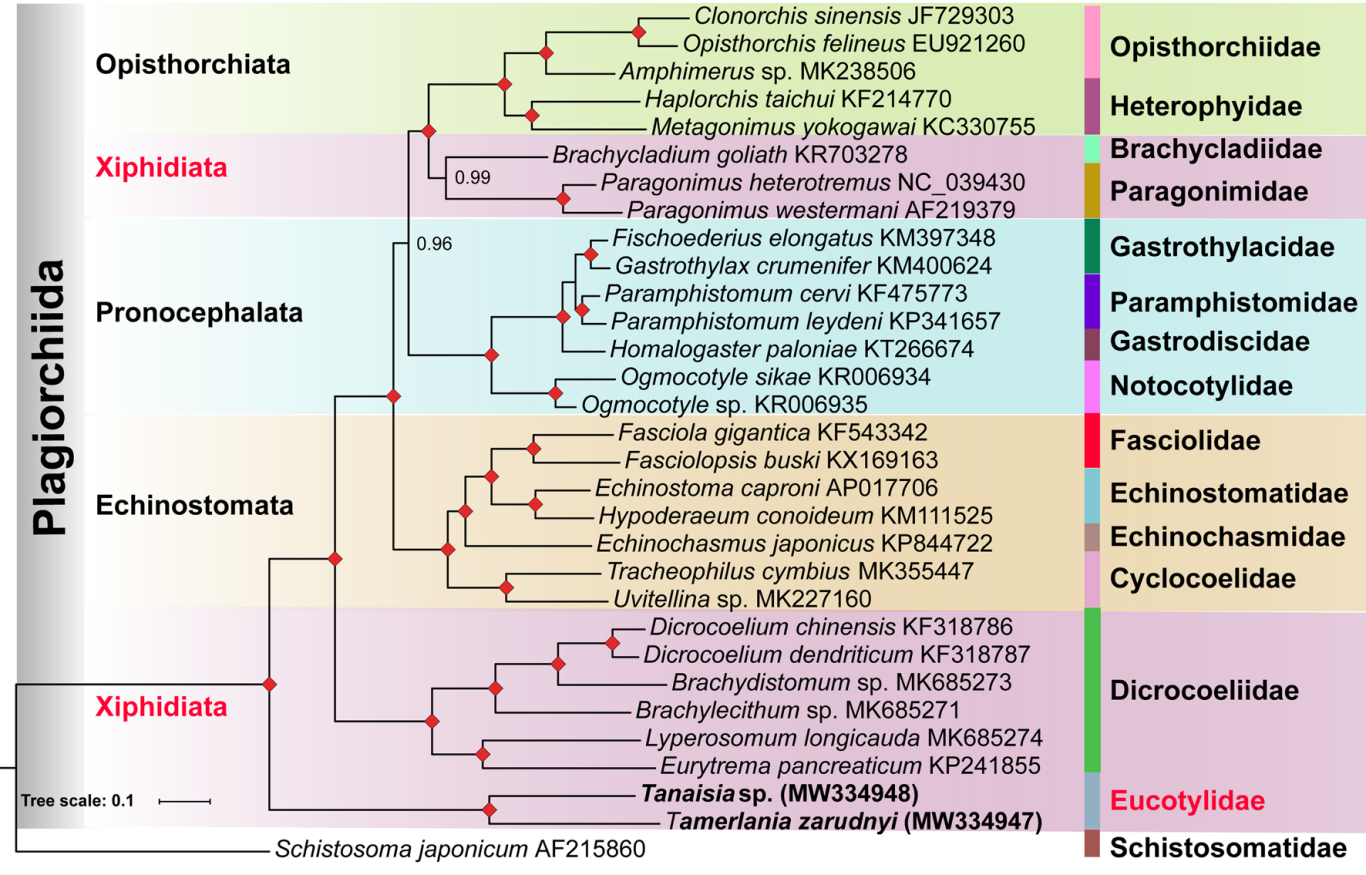
Phylogeny of the order Plagiorchiida based on Bayesian inference (BI) using concatenated amino acid sequences of mitochondrial protein-coding genes. Rectangles indicate BPP = 100; other values are given above the nodes. *Schistosoma japonicum* is the outgroup

Both ML and BI analyses (based on dataset 1) with the respective models produced phylogenetic trees with identical branch topologies and only minor differences in nodal support (Fig. 5). The BI phylogeny based on concatenated amino acid sequences of 11 PCGs (dataset 2) was consistent with that resulting from ML and BI phylogenies based on nucleotide sequences (Fig. 6). The only notable difference was the position of the clade containing members of the families of the suborder Pronocephalata Olson, Cribb, Tkach, Bray & Littlewood, 2003. In the nucleotide-based tree, it clustered together with the clade of the suborder Echinostomata (Fig. 5), while in the amino acid-based tree it formed a clade with the Opisthorchiata La Rue, 1957, and one of the clades representing paraphyletic xiphidiates (Fig. 6). This difference between phylogenies based on mitochondrial nucleotides and amino acids sequences is consistent with our previous study [26] and several other studies, including those published very recently [50].

Regardless of the dataset and model used, all phylogenies supported the paraphyletic nature of the superfamily Gorgoderoidea sensu Curran, Tkach & Overstreet, 2006, and suborder Xiphidiata. Importantly, eucotylids did not show close affinity to any other members of the Plagiorchiida included in the analysis and appeared on the trees as either a sister clade to the remaining ingroup taxa or as a member of a polytomy. Olson et al. [9] classified Paragonimidae and Dicrocoeliidae within the superfamily Gorgoderoidea based on the phylogenetic analysis using nuclear rDNA genes. However, recently published data based on 28S rDNA did not support the close relationship between the Paragonimidae and other families included in the superfamily Gorgoderoidea [12]. Our analyses suggest that the Paragonimidae might be closer to the Brachycladiidae than to the Dicrocoeliidae. The position of the Dicrocoeliidae and the Eucotylidae (Microphalloidea) outside the clade uniting other xiphidiatan trematodes (Brachycladiidae and Paragonimidae) further strengthens the suggestion expressed by Suleman et al. [26] that the content of the suborder Xiphidiata may need to be reconsidered with more sequence data.

## Conclusions

In this study, we sequenced the ITS rDNA region and 28S rDNA gene as well as complete or nearly complete mitochondrial genomes of two eucotylids, *T. zarudnyi* and *Tanaisia* sp. The complete mitochondrial genome of *T. zarudnyi* was the fourth largest mt genome of all available trematode mt genomes. The presence of a long additional string of amino acids (about 123 aa) at the 5′ end of the *cox*1 gene in mt genomes of both studied eucotylids increases the size of the *cox*1 gene, which is longer than in any of the previously sequenced trematodes and probably any flatworm. Similarly, the *rrnL* gene was longest among those reported so far from digeneans. The TΨC and DHU loops of the tRNAs varied greatly between the two eucotylids while the anticodon loop was highly conserved. Our analyses of the average sequence identity combining nucleotide diversity and Kimura-2-parameter distances between the two eucotylid mitogenomes suggested that *atp*6, *nad*5, *nad*4L and *nad*6 genes are better molecular markers for the species differentiation and population-level studies of eucotylids. Phylogenetic analyses based on mitochondrial nucleotide sequences (PCGs+RNAs) and concatenated amino acid sequences (11 PCGs) showed a lack of close relationship of the Eucotylidae with any major clade within the Plagiorchiida. Furthermore, our analyses did not support the classification of Paragonimidae and Dicrocoeliidae within the superfamily Gorgoderoidea. Similarly, the position of the Dicrocoeliidae and Eucotylidae (Microphalloidea) outside the clade uniting other xiphidiatan trematodes strengthened the proposal that the content of the suborder Xiphidiata and the interrelationships between its constituent families may need to be reconsidered.

## Supplementary information

**Additional file 1: Table S1.** Sequences of primers used to amplify and sequence the mitochondrial genomes of *Tamerlania zarudnyi* and *Tanaisia* sp.

**Additional file 2: Table S2.** Nucleotide composition and AT/GC skewness of the mitochondrial genome of *Tamerlania zarudnyi* and *Tanaisia* sp.

**Additional file 3: Figure S1.** Relative synonymous codon usage (RSCU) for the protein-coding genes of two eucotylid mitogenomes. Codon families are labeled on the x-axis. Values on the top of the bars indicate percentages of each amino acid used for the construction of protein-coding genes.

**Additional file 4: Figure S2.** Secondary structures of tRNAs (*trnH*-*trnK*) in eucotylid mitogenomes with nucleotide substitutions highlighted.

**Additional file 5: Figure S3.** Secondary structures of tRNAs (*trnS*1-*trnG*) in eucotylid mitogenomes with nucleotide substitutions highlighted, except *trn*E and *trn*G.

## Data Availability

The datasets supporting the findings of this article are included within the article and its additional files. The nuclear ITS and 28S rDNA nucleotide sequences generated in this study for the two eucotylids were deposited in the GenBank database under the accession numbers MW159308 and MW131090 for *Tamerlania zarudnyi* and MW159299 and MW139645 for *Tanaisia* sp. The mitogenomic sequences of *Tamerlania zarudnyi* and *Tanaisia* sp. are available in GenBank under the accession numbers MW334947 and MW334948, respectively.

## Abbreviations

BI: Bayesian inference
ML: Maximum likelihood
MCMC: Metropolis-coupled Markov chain Monte Carlo
PCGs: Protein-coding genes
tRNAs: Transfer RNAs
rRNAs: Ribosomal RNAs
RSCU: Relative synonymous codon usage
dN/dS: Non-synonymous/synonymous
TRs: Tandem repeats
NCRs: Non-coding regions
SNCR: Short non-coding region
LNCR: Long non-coding region
K2P: Kimura-2-parameter
