# Supplementary material for: Mitochondrial genomes of two eucotylids as the first representatives from the superfamily Microphalloidea (Trematoda) and phylogenetic implications
Source: Parasit Vectors. 2021 Jan 14;14:48. doi: 10.1186/s13071-020-04547-8 (PMC7807500; doi:10.1186/s13071-020-04547-8)
Supplement: Supplementary file 1 — Additional file 1: Table S1. Sequences of primers used to amplify and sequence the mitochondrial genomes of Tamerlania zarudnyi and Tanaisia sp. [file 13071_2020_4547_MOESM1_ESM.docx]

**Additional File 1: Table S1** Primers used for amplification and sequencing of the complete mitogenome of *Tamerlania zarudnyi* and nearly complete mitogenome of *Tanaisia* sp.

| **Fragment No.** | **Gene or region** | **Primer name** | **Sequence (5' to 3')** | **Length (bp)** |
| --- | --- | --- | --- | --- |
| F1 | CYTB | EUF1 | GCAGCCACGGTTCTTACTTC | 504 |
|  |  | EUR1 | CAAATTATAAGTAGGCAACC |  |
| F2 | CYTB-NAD4 | EUF2 | GTTTAGCTGATTCTGCAAG | 1126 |
|  |  | EUR2 | CCAAACACCAACCCCAAGC |  |
| F3 | NAD4 | EUF3 | CATTCTCCTTATTCGGAGCG | 471 |
|  |  | EUR3 | CAAAGGAACAACAGCTATGTG |  |
| F4 | NAD4-NAD1 | EUF4 | CTAATTCTTCCGGATGGTG | 2886 |
|  |  | EUR4 | CCAACAACAAGATCGAAATCC |  |
| F5 | NAD1 | EUF5 | TGCAGTTGCGTAAGGGTCC | 770 |
|  |  | EUR5 | ACAATAAGCAAACACCAAC |  |
| F6 | NAD1-COX1 | EUF6 | CTTGTGAGTATTTGATGATG | 2104 |
|  |  | EUR6 | CAGAAACACGAGAAACCCTG |  |
| F7 | COX1 | EUF7 | GTGCTGGTGTTGGATGGACG | 570 |
|  |  | EUR7 | CAATTATCATAGTCACAGAG |  |
| F8 | COX1-16S | EUF8 | CATGTGTGTATGGAGTTATC | 1927 |
|  |  | EUR8 | ACGAGTGAATCGATAAGACC |  |
| F9 | 16S | EUF9 | GGTGCAGAACCCAACGGAGG | 354 |
|  |  | EUR9 | CCTAAATCATGAACGATCC |  |
| F10 | 16S-12S | EUF10 | CAGTGGTGTAGAGGCTTCTG | 581 |
|  |  | EUR10 | GTTCTCAATAACTATGGCAAG |  |
| F11 | 12S-COX2 | EUF11 | CTGTAAGCTGGTCCCTTCTG | 608 |
|  |  | EUR11 | CAAGATTCAAGTACGCTCCAC |  |
| F12 | 12S-COX2 | EUF12 | GTAGGTTGGGATTAGTTAGCC | 531 |
|  |  | EUR12 | CAATCAACGAATGCGGAATCC |  |
| F13 | COX2 | EUF13 | CAATGCATTGGGTTGATAGTG | 312 |
|  |  | EUR13 | CTGCACCACACAACTCACTAC |  |
| F14 | COX2-CYTB | EUF14 | CTGCTTTTCATCTTAAGCTGG | 5666 |
|  |  | EUR14 | GAGCAGAATAAGCACGAGAC |  |
