# Supplementary material for: Mitochondrial genomes of two eucotylids as the first representatives from the superfamily Microphalloidea (Trematoda) and phylogenetic implications
Source: Parasit Vectors. 2021 Jan 14;14:48. doi: 10.1186/s13071-020-04547-8 (PMC7807500; doi:10.1186/s13071-020-04547-8)
Supplement: Supplementary file 2 — Additional file 2: Table S2. Nucleotide composition and AT/GC skewness of the mitochondrial genome of Tamerlania zarudnyi and Tanaisia sp. [file 13071_2020_4547_MOESM2_ESM.docx]

**Additional file 2: Table S2** Nucleotide composition and AT/GC skewness of the mitochondrial genome of *Tamerlania zarudnyi* and *Tanaisia* sp.

| Regions | Size (bp) | T(U) | C | A | G | AT (%) | GC (%) | AT skewness | GC skewness |
| --- | --- | --- | --- | --- | --- | --- | --- | --- | --- |
| *Tamerlania zarudnyi*/*Tanaisia* sp. | | | | | | | | | |
| Full genome | 16188/13953 | 41.9/41.5 | 12.2/13.6 | 18.7/15.2 | 27.2/29.7 | 60.6/56.7 | 39.4/43.3 | -0.384/-0.464 | 0.38/0.371 |
| PCGs | 10677/10176 | 45.7/44.3 | 11.9/13.1 | 15.4/13.3 | 27/29.3 | 61.1/57.6 | 38.9/42.4 | -0.496/-0.538 | 0.388/0.383 |
| tRNAs | 1492/1335 | 34.7/32.2 | 13.9/15.8 | 22.3/20.8 | 29/31.2 | 57/53 | 42.9/47 | -0.217/-0.215 | 0.351/0.327 |
| rRNAs | 2002/2008 | 33.3/32.9 | 14.5/15.4 | 24/21.5 | 28.2/30.2 | 57.3/54.4 | 42.7/45.6 | -0.163/-0.209 | 0.319/0.323 |
| 1st codon position | 3559/3392 | 39.3/39.7 | 12.6/12.9 | 19.2/17.2 | 28.9/30.2 | 58.5/56.9 | 41.5/43.1 | -0.344/-0.394 | 0.392/0.399 |
| 2nd codon position | 3559/3392 | 45.7/44.8 | 15.5/15.7 | 17.1/16.4 | 21.7/23.1 | 62.8/61.2 | 37.2/38.8 | -0.454/-0.464 | 0.167/0.189 |
| 3rd codon position | 3559/3392 | 52.3/48.6 | 7.6/10.5 | 9.9/6.4 | 30.3/34.6 | 62.2/55 | 37.9/45.1 | -0.682/-0.768 | 0.601/0.535 |
| *atp*6 | 537/534 | 47.5/43.4 | 13.4/14.4 | 16.4/16.1 | 22.7/26 | 63.9/59.5 | 36.1/40.4 | -0.487/-0.459 | 0.258/0.287 |
| *cox*1 | 2055/2061 | 42/41.1 | 14/14.6 | 17.6/15.1 | 26.4/29.2 | 59.6/56.2 | 40.4/43.8 | -0.411/-0.462 | 0.308/0.335 |
| *cox*2 | 597/600 | 43.2/42.5 | 11.7/13 | 16.8/15.7 | 28.3/28.8 | 60/58.2 | 40/41.8 | -0.441/-0.461 | 0.414/0.378 |
| *cox*3 | 651/120 | 49.9/46.7 | 10.3/8.3 | 13.8/17.5 | 26/27.5 | 63.7/64.2 | 36.3/35.8 | -0.566/-0.455 | 0.432/0.535 |
| *cytb* | 1113/1113 | 46.3/42.2 | 10.6/13.8 | 15.5/13.6 | 27.6/30.4 | 61.8/55.8 | 38.2/44.2 | -0.497/-0.514 | 0.445/0.374 |
| *nad*1 | 897/918 | 47.9/47.2 | 9.1/10 | 14.5/11.3 | 28.4/31.5 | 62.4/58.5 | 37.5/41.5 | -0.536/-0.613 | 0.513/0.517 |
| *nad*2 | 870/867 | 47/49.4 | 13.6/11.4 | 15.1/10.3 | 24.4/29 | 62.1/59.7 | 38/40.4 | -0.515/-0.656 | 0.285/0.434 |
| *nad*3 | 357/351 | 50.4/48.4 | 8.1/10.3 | 13.7/10.5 | 27.7/30.8 | 64.1/58.9 | 35.8/41.1 | -0.572/-0.643 | 0.547/0.5 |
| *nad*4 | 1284/1305 | 45.9/43.9 | 11.6/13.9 | 14.8/14.5 | 27.7/27.7 | 60.7/58.4 | 39.3/41.6 | -0.512/-0.504 | 0.41/0.33 |
| *nad*4L | 270/273 | 47.8/46.2 | 9.3/10.3 | 13.3/14.7 | 29.6/28.9 | 61.1/60.9 | 38.9/39.2 | -0.564/-0.518 | 0.524/0.477 |
| *nad*5 | 1596/1587 | 44.4/44.6 | 12.1/13.5 | 14.1/11.5 | 29.4/30.4 | 58.5/56.1 | 41.5/43.9 | -0.518/-0.591 | 0.418/0.383 |
| *nad*6 | 450/447 | 49.3/48.1 | 13.1/12.8 | 16/11.6 | 21.6/27.5 | 65.3/59.7 | 34.7/40.3 | -0.51/-0.61 | 0.244/0.367 |
| *rrnL* | 1244/1231 | 35.9/35.8 | 13.1/13.6 | 22.2/20.1 | 28.9/30.4 | 58.1/55.9 | 42/44 | -0.235/-0.28 | 0.375/0.38 |
| *rrnS* | 758/777 | 29.2/28.2 | 16.9/18.3 | 26.9/23.7 | 27/29.9 | 56.1/51.9 | 43.9/48.2 | -0.04/-0.087 | 0.231/0.241 |

Abbreviations: bp, base pairs; PCGs, protein-coding genes
