# Supplementary figures and images for: Mitochondrial genomes of two eucotylids as the first representatives from the superfamily Microphalloidea (Trematoda) and phylogenetic implications
Source: Parasit Vectors. 2021 Jan 14;14:48. doi: 10.1186/s13071-020-04547-8 (PMC7807500; doi:10.1186/s13071-020-04547-8)

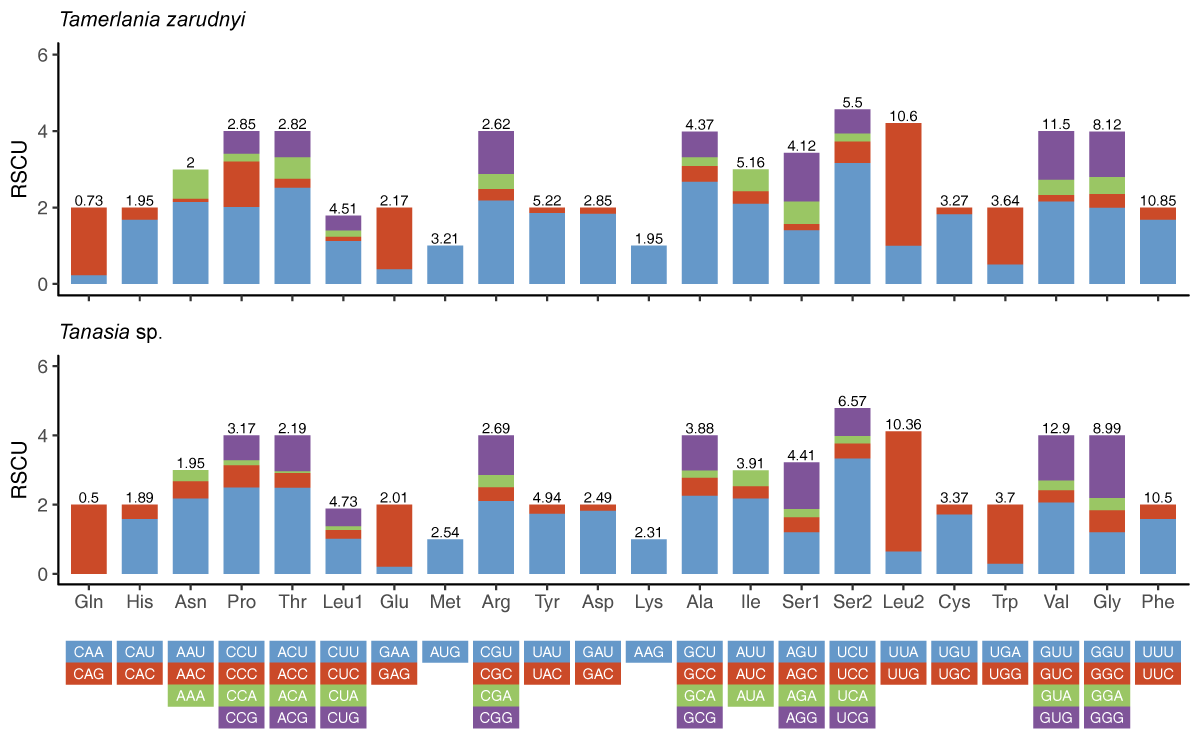

Supplement: Supplementary file 3 — Additional file 3: Figure S1. Relative synonymous codon usage (RSCU) for the protein-coding genes of two eucotylid mitogenomes. Codon families are labeled on the x-axis. Values on the top of the bars indicate percentages of each amino acid used for the construction of protein-coding genes. [file 13071_2020_4547_MOESM3_ESM.tif]

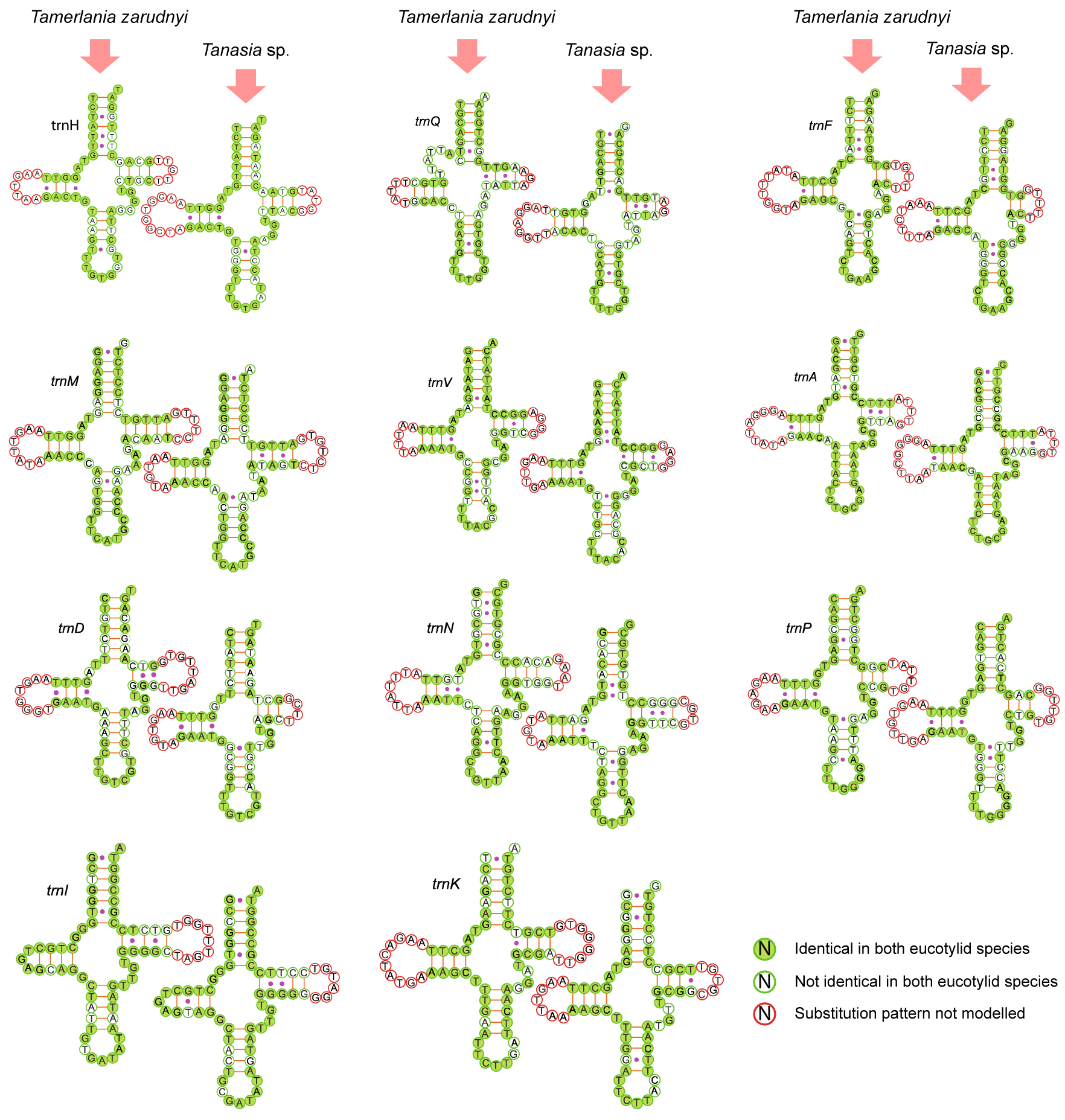

Supplement: Supplementary file 4 — Additional file 4: Figure S2. Secondary structures of tRNAs (trnH-trnK) in eucotylid mitogenomes with nucleotide substitutions highlighted. [file 13071_2020_4547_MOESM4_ESM.tif]

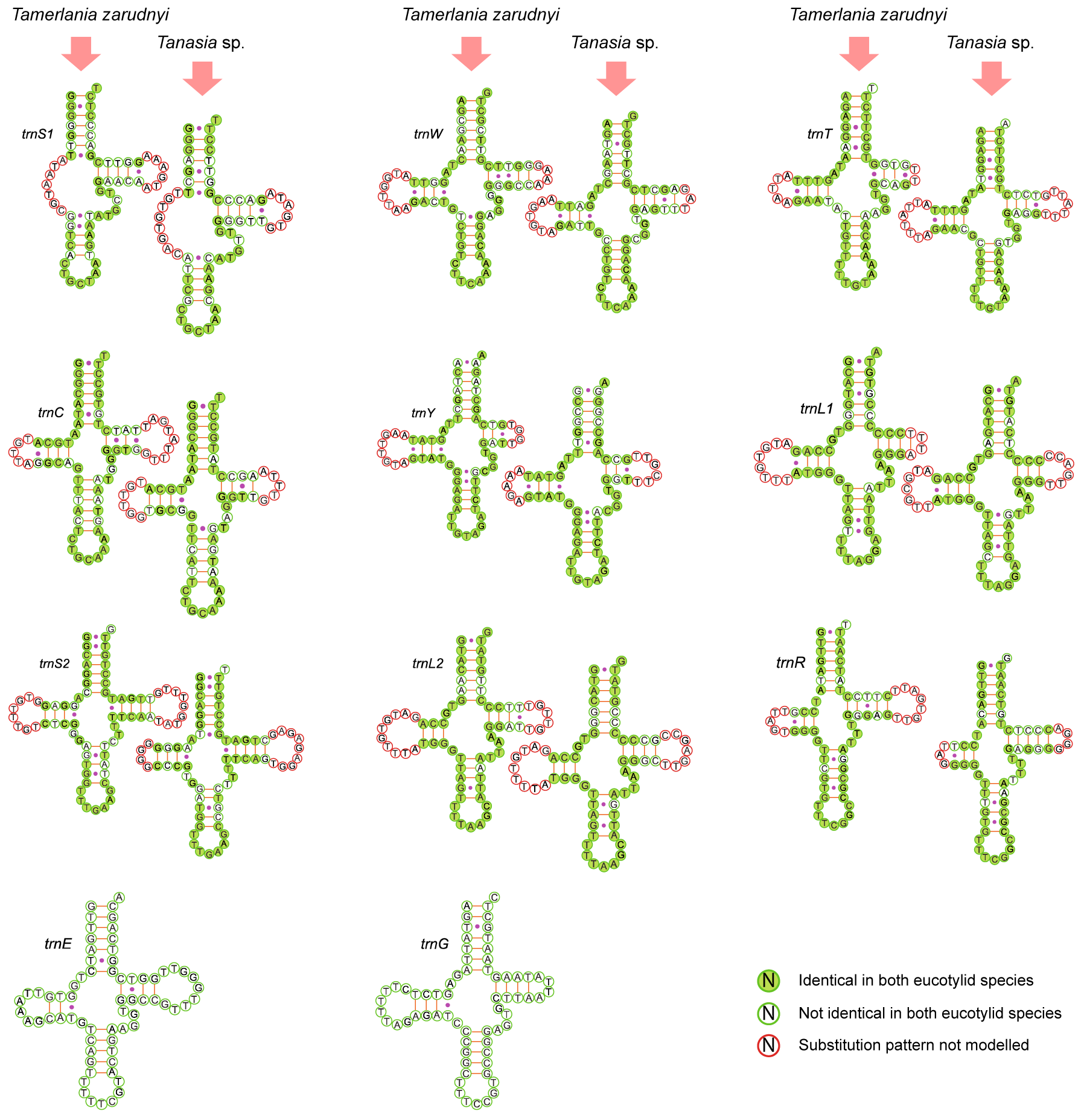

Supplement: Supplementary file 5 — Additional file 5: Figure S3. Secondary structures of tRNAs (trnS1-trnG) in eucotylid mitogenomes with nucleotide substitutions highlighted, except trnE and trnG. [file 13071_2020_4547_MOESM5_ESM.tif]
